# Simultaneous determination of meloxicam and bupivacaine via a novel modified dual wavelength method and an advanced chemometric approach

**DOI:** 10.1038/s41598-024-51885-z

**Published:** 2024-01-22

**Authors:** Samah F. El-Malla, Aliaa A. Hamza, Samar H. Elagamy

**Affiliations:** Department of Pharmaceutical Analytical Chemistry, Faculty of Pharmacy, Tanta University, Tanta, Egypt

**Keywords:** Analytical chemistry, Green chemistry

## Abstract

This study presents two spectrophotometric methods; a novel dual wavelength—derivative spectrophotometry and multivariate curve resolution-alternating least squares (MCR-ALS) for the simultaneous determination of a fixed dose combination of bupivacaine (BUP) and meloxicam (MEL) in a ratio of 30:1. The extended UV spectrum of MEL enables its direct determination at λ_max_ 360 nm with no interference from BUP. The determination of BUP was unfeasible directly because the UV spectra of both drugs are moderately overlapped over the wavelength range of 250–450 nm, thus new chemometric based spectrophotometric methods should be developed for its determination. Dual wavelength-derivative method was employed based on using first derivative spectra. The selected dual wavelengths for determination BUP were 274.6 nm and 374.6 nm where the dA/dλ amplitudes differences for MET are equal to zero. MCR-ALS is advanced chemometric tool that enables analysis of multicomponent samples in complex matrices with high resolution based on the decomposition of signal/spectral data into the pure spectra and corresponding concentration profile. The figures of merits for MCR model show that there is a good agreement between the actual and predicted concentrations for MEL and BUP. The methods were validated and statistically compared with a reported HPLC method.

## Introduction

**Figure 1:**
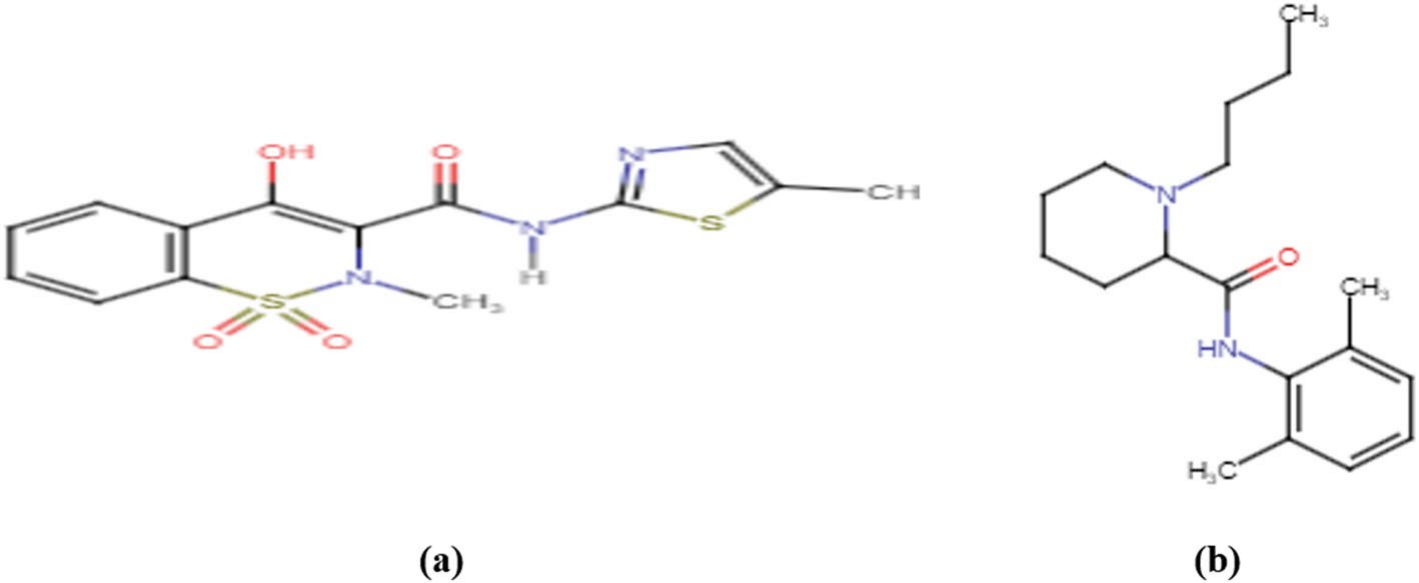
Chemical structure of MEL (**a**) and BUP (**b**).

Meloxicam(MEL);[4-hydroxy-2-methyl-*N*-(5-methyl-1,3-thiazol-2-yl)-2H-1,2-benzothiazine-3-carboxamide-1,1-dioxide] is a non-steroid anti-inflammatory (NSAID) drug that inhibits cyclo-oxygenase II(COX-II) resulting in decreasing the conversion of arachidonic acid into prostaglandin precursors Fig. 1a. MEL is typically administered in rheumatic diseases and osteoarthritis^1–3^.

MEL is soluble in organic solvents such as DMSO and DMF and it’s practically insoluble in water^4^.

Bupivacaine (BUP); [1-butyl-*N*-(2,6-dimethylphenyl)piperidine-2-carboxamide] is a long acting local anesthetic Fig. 1b. The mechanism of action is based on binding to sodium ion channels in neuronal membrane that in turn decreases the voltage-dependent membrane permeability to sodium ions, depolarization and conduction of nerve impulse which results in relieving the pain^1,2,5^. BUP is soluble in water and different organic solvents such as ethanol, DMSO, and DMF^6^.

The fixed dose MEL and BUP combination (ZYNRELEF®) is a newly introduced postsurgical analgesic in the market that works for up to 72 h^7^.

The literature review reveals different chromatographic methods for simultaneous determination of both drugs including UPLC-MS/MS^8^, RP-HPLC and HPTLC^9^. Few spectrophotometric methods have been reported for determination of such combination as fourier self-deconvolution, ratio difference, first derivative and ratio first derivative spectrophotometry^10^.

The simultaneous determination of pharmaceutical multicomponents requires analytical methods that are simple, cost-effective, reproducible, and suitable for routine quality control. HPLC is the primary technique for pharmaceutical analysis, offering excellent separation and selectivity. However, drawbacks such as solvent consumption, time-intensive procedures, high instrument costs, and the need for expertise limit its accessibility. To address these challenges, UV spectrophotometry serves as a viable and green substitute for HPLC. While many pharmaceuticals exhibit suitable light absorption in the UV region, the overlapping spectra of some drugs make direct analysis challenging. In overcoming this limitation, various chemometric methods have been applied to resolve the overlapped spectra. Since the UV spectra of BUP and MEL are overlapped, this study aims to develop simple spectrophotometric methods for simultaneous determination of BUP and MEL. The methods are dual wavelength-derivative spectrophotometry (DWD) and multivariate curve resolution-alternating least squares (MCR-ALS). DWD is a novel method which involves modification of the well-established dual wavelength method. The method is useful for determination of binary mixture where there are no points in their zero order spectra that can enable application of dual wavelength principle^11^. In this case, the first derivative spectra is utilized and the selection of the two wavelength for measurement relies on finding out the points where dA/dλ for one component is zero at while that for the other is significant and is directly proportional to its concentration. MCR-ALS is a chemometric tool that allows extraction of spectra data and concentration profile of pure components in multicomponent mixtures in complex matrices. MCR-ALS enables quantitative analysis of various analytes such as agricultural samples, pharmaceuticals, veterinary drugs, and environmental samples^12–14^. The developed methods enable simple and rapid simultaneous determination of diluted samples containing both drugs.

## Experimental

### Apparatus

Shimadzu (Japan) UV-1800 PC double beam spectrophotometer with 1cm Quartz cells was used for spectrophotometric measurements with UV probe software for calculations and displaying spectra. Zero-order UV spectra in range from 250 to 450 nm at 0.1 nm sampling interval with scanning speed 400 nm s^−1^ were recorded, MCR-ALS was performed using Unscrambler® X software.

### Materials

MEL (99%, purity), BUP (99%, purity) and dosage form inactive components as dimethyl sulfoxide, maleic acid, triethylene glycol and triacetin were kindly supplied from ADWIA Company for Pharmaceutical Industries, 10th of Ramadan, Cairo, and sodium deocyl sulfate (SDS) from sigma Aldrich company, Cairo, Egypt.

### Preparation of standard solutions

Standard stock solution of 1.0 mg mL^−1^ of BUP was prepared by dissolving 100 mg of BUP in 0.3% SDS in a 100-mL volumetric flask. Then different aliquots (1.0–9.0 mL) were diluted with the same solvent in 10-mL volumetric flasks to get working standards at the concentration range of 100–900 μg mL^−1^ in MCR method while in DWD method taking different volumes (0.7–9.0 mL) were taken and diluted in 10-mL volumetric flasks for preparing standard solutions at concentrations from 70 to 900 μg mL^−1^. The standard stock solution of MET (1.0 mg mL^−1^) was prepared by the same procedure as BUP then different aliquots (0.2–1.2 mL) were diluted with 0.3% SDS in 10-mL volumetric flasks to get calibration standards in the final concentration of 20–120 μg mL^−1^.

### Preparation of calibration and validation sets for MCR-ALS

A set of 20 calibration mixture were prepared with variable ratio of BUP and MEL. A validation set of another 5 mixture solutions containing concentrations differ from those for calibration model was also prepared in triplicate times. Table S1 show the concentrations of BUP and MEL in the calibration and validation sets.

### Construction of calibration curve for DWD method

The calibration curve of BUP was constructed by plotting the difference of dA/dλ amplitudes at 274.6 nm and 374.6 nm against the concentrations from 70 to 900 μg mL^−1^ showing great difference values for BUP detection with no difference in case of MEL, while the calibration curve of MEL was established at its zero order spectra by plotting absorbance at 360 nm versus concentrations from 20–120 μg mL^−1^.

### Assay of Lab prepared synthetic mixture

ZYNRELEF® is single dose vial 2.0 mL formulation containing 30 mg mL^−1^ of BUP and 1.0 mg mL^−1^ of MEL Synthetic mixture was prepared by accurate weighting 300 mg BUP, 10 mg MEL, 0.59 mg maleic acid, 730 mg triethylene glycol, and 293 mg triacetin. All ingredients were dissolved in 5 mL dimethyl sulfoxide (DMSO) in 10-mL volumetric flask, sonicated for 15 min, then completed to volume with DMSO to get stock solution. 2.5 mL from the stock was taken and diluted with 0.3% SDS in 100-ml volumetric flask. The concentrations of BUP and MEL were calculated from the derived equation of the constructed calibration curves.

## Results and discussion

Achieving complete solubility for MEL and BUP in the same solvent was challenging as various trials were conducted for finding a suitable, environmentally friendly solvent that would achieve the complete solubility of both drugs. Initially, DMSO (dimethyl sulfoxide) was employed and demonstrated its effectiveness in completely dissolving both MEL and BUP. However, concerns arise regarding the chronic toxicity associated with high concentrations of DMSO through contact, ingestion, or inhalation^15^. Consequently, an alternative, SDS (sodium dodecyl sulfate), was tested and found to achieve complete solubility while presenting fewer hazards than DMSO^16^.

Meloxicam exhibits lipophilic properties and is known for its limited solubility in water. The enhanced solubility of meloxicam in SDS can be primarily attributed to the hydrophobic interactions that occur between meloxicam molecules and the hydrophobic alkyl chains in SDS^17,18^. Whereas BUP has a pKa of 8.09, and it can exist in an aqueous solution as positively charged form with a protonated cyclic tertiary amine. Consequently, the solubility of BUP in SDS can be rationalized to electrostatic interactions between the cationic group in BUP and the anionic sulfate groups in SDS^19^. In order to optimize the solubility, various concentrations of SDS above its critical micelle concentration (CMC) were prepared. It was found that a concentration of 0.3% SDS yielded the highest absorbance for both drugs Fig. S1.

**Figure 2:**
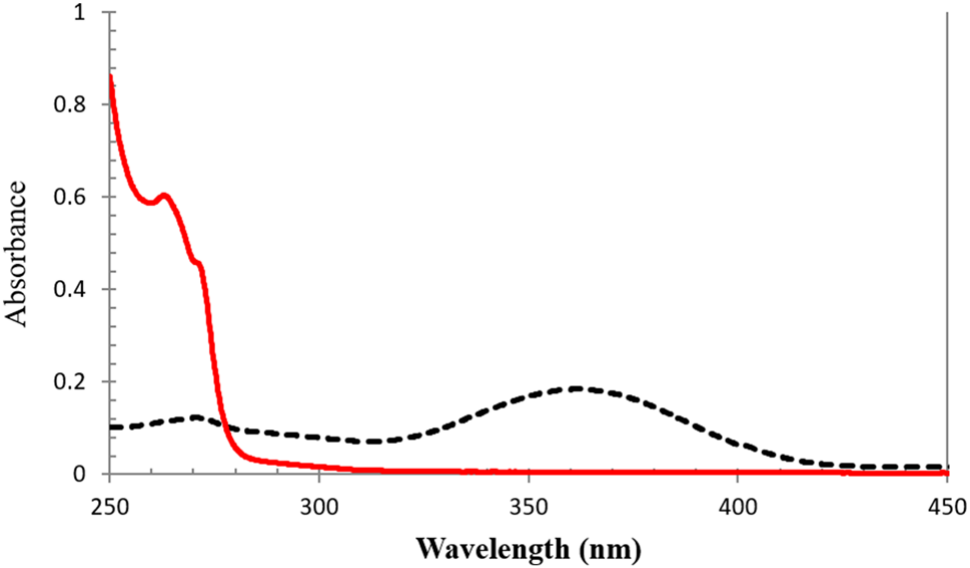
Zero order UV absorption spectra of 25 μg mL^−1^ MEL (black) and 750 μg mL^−1^ BUP (red) in 0.3% SDS.

The UV spectra of MEL and BUP shows overlapping over the wavelength range of 250–450 nm Fig. 2. To address this issue, two mathematical-based methods, namely dual wavelength-derivative (DWD) and multivariate curve resolution-alternating least squares (MCR-ALS), were selected for their simplicity and suitability for simultaneous determination of BUP and MEL in our study.

### Theories of the developed methods

#### Dual wavelength-derivative spectrophotometry (DWD)

**Figure 3:**
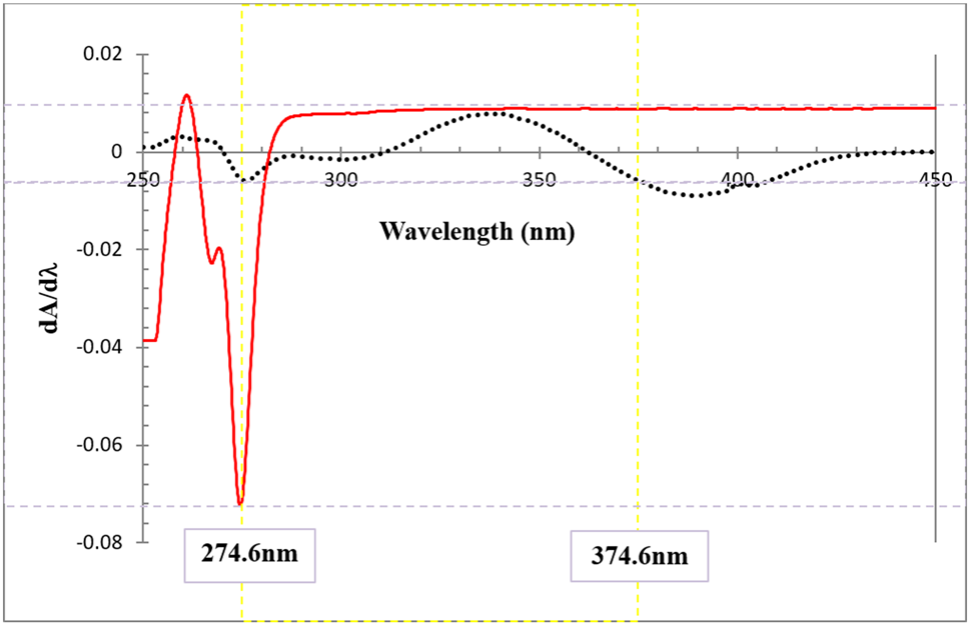
First derivative UV absorption spectra of 40 μg mL^−1^ MEL (black) and 700 μg mL^−1^ BUP (red) in 0.3% SDS showing wavelength selection in DWD method for determination of BUP.

The traditional dual wavelength method DW relies on determination of the unknown concentration of a specific component within a binary mixture through calculating the absorbance difference between two points on the mixture's zero order spectra. To utilize this method, it is essential to carefully select two wavelengths where the interfering component exhibits identical absorbance, while the component of interest shows a noticeable absorbance difference. Sometimes the selection of such wavelengths is not applicable as in the case of the spectra of BUP and MEL Fig. 2 thus, dual wavelength derivative DWD method was applied on their first derivative spectra where the wavelengths at 274.6 nm and 374.6 nm have significant dA/dλ differences for BUP and negligible difference for MEL which enable quantitative determination of BUP with no interference from MEL. Figure 3 shows the application of dual wavelength derivative for determination of BUP. This work represents a novel approach for simultaneous determination of drug mixtures based on the assumption that principles valid in zero-order are applicable in the first derivative, and it also offers sensitivity and simplicity.

#### Multivariate curve resolution-alternating least squares MCR-ALS

MCR decomposes the data matrix to extract the most relevant data of pure components in a mixture. This model is expressed as:

1 $$D = CS^{T} + E$$

where (*D*) is the data matrix that contains the spectra of the drug mixture of interest. (*C*) is the concentration matrix of pure components, and (*S*^T^) is the pure spectra matrix. (*E*) is the matrix of residuals (the data which cannot be identified by the model such as experimental errors).

The iterative alternating least squares (ALS) procedure is performed. This iteration process involves decomposition of the (D) matrix (spectral data) into bilinear relation between the data matrix and components concentrations, estimation of the optimum number of components, and finally the determination of the concentration matrix and pure spectra matrix (*C* and *S*^T^)^20,21^. Pure spectra of 500 μg mL^−1^ BUP and 50 μg mL^−1^ MEL were applied for initial estimations for the constructed MCR-ALS model. The constraints employed in constructing the MCR-ALS model included non-negative constraints on both the concentrations of the chemical constituents and their corresponding spectra. The model was utilized to predict the concentrations of BUP and MEL in calibration and validation set. To evaluate the effectiveness of the proposed MCR-ALS model, various performance metrics, including Root Mean Square Error of Prediction (RMSEP), bias, and Standard Error of Prediction (SEP), were calculated using the corresponding equations:

$$RMSEP=\sqrt{\frac{{\sum }_{i=1}^{n}{\left({c}_{i}-\widehat{{c}_{i}}\right)}^{2}}{n}}$$

$$SEP=\sqrt{\frac{{\sum }_{i=1}^{n}{\left({c}_{i}-\widehat{{c}_{i}}-Bias\right)}^{2}}{n-1}}$$

$$Bias=\frac{{\sum }_{i=1}^{n}\left({c}_{i}-\widehat{{c}_{i}}\right)}{n}$$

where (cὶ) is the known concentration of analyte and (ĉ ὶ) is the predicted concentration, n is the total number of samples forming the calibration or validation set.

The results for calibration and validation model were listed in Table 1. The calibration model of BUP and MEL was established in the concentration range of 100–900 and 20–120 µg mL^−1^, respectively.

**Table 1:** Figures of merit of the MCR-ALS regression model and concentrations for calibration and validation.

Parameters	Calibration model		Validation model	
	BUP	MEL	BUP	MEL
Slope	0.005373	0.030305	0.0024136	0.0418173
Intercept	0.047911	0.119799	0.3873297	0.3821937
RMSEP^a^ (µg mL^−1^)	10.524	3.6766	13.469837	1.4017469
SEP^b^ (µg mL^−1^)	11.09369	3.8755	17.161663	1.4985305
Bias	0.001418	− 2.9 × 10^−5^	0.0042725	− 6.79 × 10^−5^
Correlation	0.996	0.986	0.96	0.997

^a^Root mean square error of prediction.

^b^Standard error of prediction.

### Validation of the proposed methods

Proposed methods were validated regarding linearity, specificity, accuracy, repeatability, and intermediate precision according to ICH Q2 (R1)^22^.

#### Linearity

**Figure 4:**
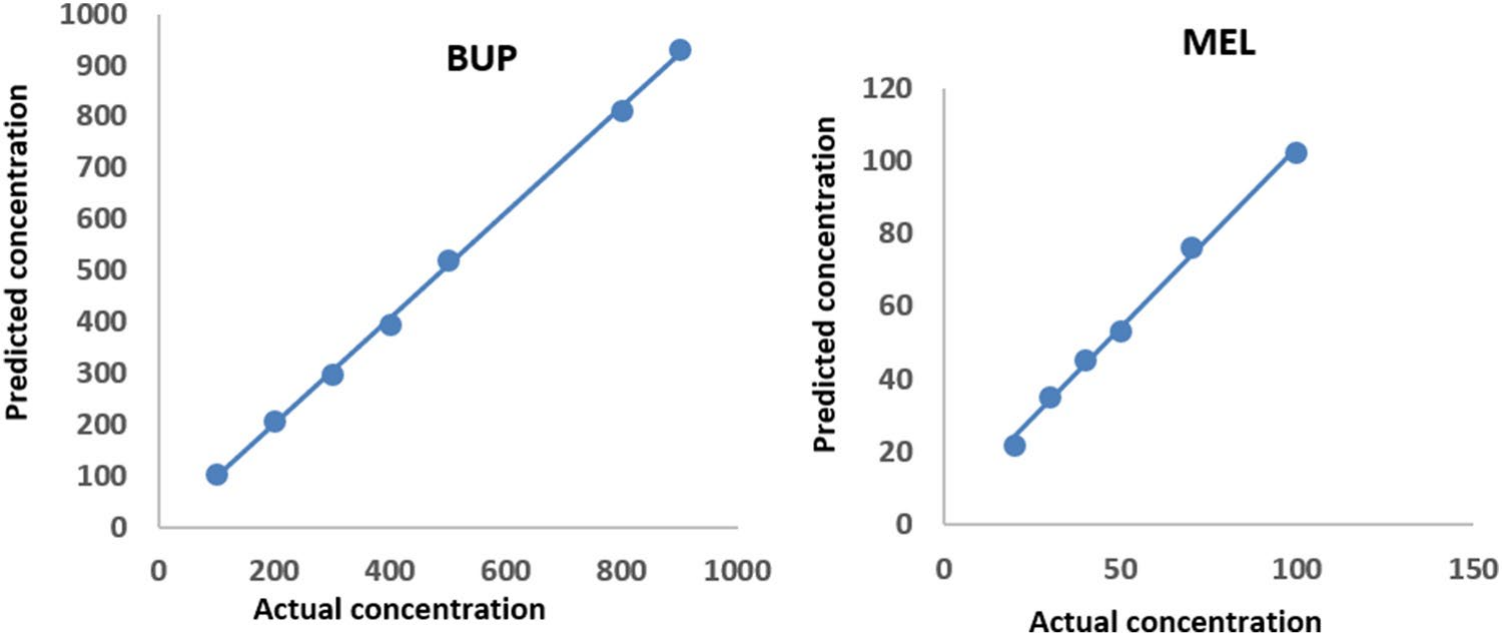
The plots of actual conc. vs. predicted conc. of MET and BUP for calibration models.

The calibration graphs constructed by plotting the values of response (absorbance, ∆ [dA/dλ]) against drug concentrations (μg mL^−1^) were linear over the range (100–900 μg mL^−1^) for BUP and (20–120 μg mL^−1^) for MEL. In MCR-ALS method, the calibration graphs for BUP and MEL were constructed between predicted versus actual concentrations. The results show that there is a good agreement between the predicted and actual values (Fig. 4). A linear regression fit was conducted to correlate the known and predicted concentrations, from which the slope, intercept, limit of detection (LOD), and limit of quantification (LOQ) were determined. The regression parameters for all proposed methods were listed on Table 2

**Table 2:** The regression parameters for direct spectrophotometry for MEL and DWD for BUP.

Method	Direct spectrophotometry (MEL)	DWD (BUP)	MCR-ALS	
			MEL	BUP
Wavelength (nm)	360	274.6–374.6	–	–
Concentration range (μg mL^−1^)	20–120	70–900	20–120	100–900
Limit of detection LOD (μg mL^−1^)	2.39	13.2	5.64	22.78
Limit of quantitation LOQ (μg mL^−1^)	7.25	40	17.12	69.02
Regression parameters				
Slope ± SD (S_b_)	0.0089 ± 8.29E − 05	0.0001 ± 8.65E − 07	0.9934 ± 0.029	1.0301 ± 0.013
Intercept ± SD (S_a_)	0.0062 ± 0.0064	− 0.0004 ± 0.0004	4.1751 ± 1.7006	− 5.0315 ± 7.11
SD of residual (S_y/x_)	0.0069	0.0009	1.91	9.75
Correlation coefficient (r)	0.9997	0.9993	0.9978	0.9992

#### Limit of quantitation and limit of detection

LOQ and LOD are calculated by the following equations respectively:

$$\begin{aligned} & {\text{LOQ}} = 10\;\upsigma {\text{/S}}. \\ & {\text{LOD}} = 3.3\;\upsigma {\text{/S}}. \\ \end{aligned}$$

where σ is the standard deviation of intercept, and S is the slope of the calibration curve.

##### Accuracy

In order to assess the accuracy of the proposed methods, triplicate analyses of binary mixtures MEL and BUP were conducted across their respective linearity ranges. The accuracy was validated by computing the mean percentage recovery along with the standard deviation (± SD). The results, which were presented in Table 3, indicate that the mean percentage recovery for both drugs fell within the range of 98–102%, demonstrating the accuracy of the developed methods.

**Table 3:** The results for accuracy for the proposed methods.

Method for determination of drug	Ratio of drug in mixture (MEL:BUP)	Concentration taken (μg mL^−1^)	Mean concentration found* (μg mL^−1^)	% recovery	Mean % recovery ± SD
Direct spectrophotometry for determination of (MEL)	25:750	25	25.18	100.72	100.45 ± 1.23
	60:300	60	60.92	101.53	
	100:100	100	99.10	99.10	
DWD for determination of (BUP)	25:750	750	736.5	98.2	99.79 ± 0.54
	60:300	300	297.8	99.27	
	100:100	100	98.9	98.9	
MCR-ALS for determination of (MEL)	50:200	50	50.29	100.58	100.203 ± 0.87
	40:300	40	40.88	102.2	
	80:500	80	80.67	100.83	
MCR-ALS for determination of (BUP)	50:200	200	203.56	101.87	100.97 ± 1.4
	40:300	300	297	99.33	
	80:500	500	508.66	101.733	

*Average of triplicate measurement.

#### Precision

Precision was assessed in two aspects: repeatability and intermediate precision. Repeatability was evaluated by performing triplicate determinations of binary mixtures of both drugs within the same day. On the other hand, intermediate precision was evaluated by repeating the determinations of BUP and MEL for three consecutive days. The precision of the developed methods was confirmed by observing low values of relative standard deviation (% RSD) below 2.0%, confirming the reliability of the developed methods. The results are listed in Table 4.

**Table 4:** The results for precision of the proposed methods.

Method for determination of drug	Ratio of drug in mixture (MEL:BUP)	Concentration taken (μg mL^−1^)	Intra-day precision		Inter-day precision	
			Mean concentration found* (μg mL^−1^)	% RSD	Mean concentration found* (μg mL^−1^)	% RSD
Direct spectrophotometry for determination of (MEL)	25:750	25	25.31	0.47	25.37	0.55
	60:300	60	60.97	0.09	60.51	0.66
	100:100	100	99.2	1.16	100.92	1.51
DWD for determination of (BUP)	25:750	750	735.93	0.1	742.6	0.79
	60:300	300	297.57	0.23	298.48	0.94
	100:100	100	99.3	0.53	99.63	0.67
MCR-ALS for determination of (MEL)	50:200	50	50.3	0.66	50.4	0.86
	40:300	40	40.19	0.98	40.9	0.98
	80:500	80	80.6	0.56	81.6	0.56
MCR-ALS for determination of (BUP)	50:200	200	203.5	1.82	200.9	1.82
	40:300	300	295.76	1.56	305.1	1.56
	80:500	500	501.66	1.03	504.66	1.03

*Average of triplicate measurement.

#### Specificity and interference

The proposed methods were successfully employed to simultaneously determine the concentrations of BUP and MEL in a laboratory-prepared mixture. The calculated mean percentage recovery values fell within an accepted range of 100% ± 10%, indicating that there were no interference from the excipients. Furthermore, the results were subjected to statistical comparison with a previously reported HPLC–DAD method by analyzing the average peak areas of six determinations of the drugs at a detection wavelength of 260.5 nm^9^. The t-test and F-test results showed that both methods exhibited no significant differences, as the calculated t- and F-values were less than the theoretical values, confirming the accuracy and precision of the developed methods. The results are summarized in Table 5.

**Table 5:** Statistical comparison of the proposed methods with the reported HPLC method.

Method	Drug	Concentration taken (μg mL^−1^)	Mean concentration found* (μg mL^−1^)	Proposed methods	Reported method (9)	t-value	F-value
				Mean % recovery* ± SD		(2.228)^a^	(5.05)^b^
Direct	MEL	25	25.133	100.67 ± 1.19	99.88 ± 1.06	1.2	1.27
MCR-ALS	MEL	25	25.4149	101.37 ± 1.31		2.17	1.27
MCR-ALS	BUP	750	740.889	98.99 ± 0.62	99.81 ± 1.62	1.15	4.89
DWD	BUP	750	759.833	101.31 ± 0.85		2	3.63

ZYNRELEF® contains 30 mg mL^−1^ BUP and 1 mg mL^−1^ MEL.

*Average of six determinations.

^a,b^Theoretical values for Student’s t-test and F test.

### Greenness of the proposed method in comparison with the reported method

**Figure 5:**
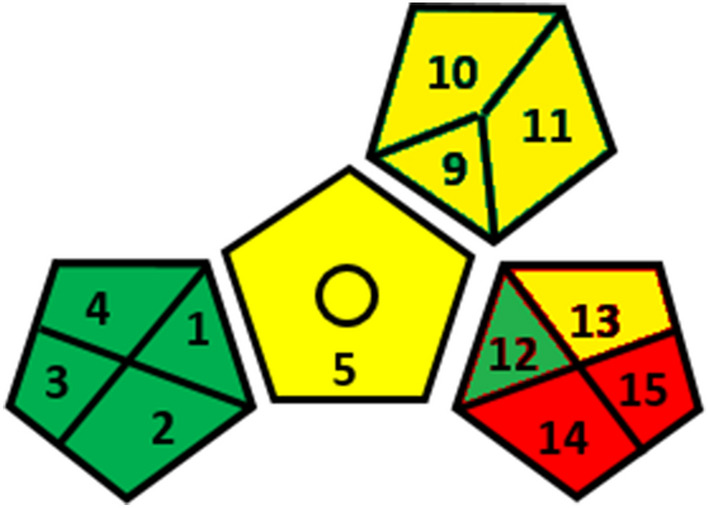
GAPI model for the developed methods.

The greenness of the developed methods was evaluated using two common tools, analytical eco-scale and green analytical procedure index (GAPI). The output of analytical eco-scale has a numerical value indicating the greenness of the method through deduction of the penalty points (PP) from 100^23^. The analytical eco-score for the proposed methods is 90 indicating an excellent green method Table 6. The output of GAPI is based on color representation indicating the environmental impact of all steps of the analytical methods^24,25^. As shown in Fig. 5, the procedures outlined in the methods eliminate the need for sample preparation or extraction steps, leading to the coloration of sections 1–4 in green on the GAPI diagram and sections 6–8 have been excluded from the diagram. Section 5 is highlighted in yellow, indicating a simple procedure requirement. SDS reagent was utilized in all steps of the methods, and it is associated with mild health and safety hazard resulting in the coloration of sections 10–11 in yellow. Also, the reagent and solvents volume is 10–100 mL, thus Section 9 is marked in yellow. The spectrophotometer's low energy consumption of less than 0.1 kWh per sample is reflected in the green coloration of section 12. It is noteworthy that the proposed methods do not involve hermetic sealing or the emission of hazardous vapors, hence section 13 is colored in yellow. The only sections colored in red are 14–15, denoting a waste volume is 10 mL without a specified waste treatment method. Table 7 shows a comparison of the developed methods with other reported methods in terms of linearity, limits of detection, and greenness assessment, it is evident that the developed methods exhibit a superior green assessment when compared to the other reported methods.

**Table 6:** The penalty points of UV and chemometric spectrophotometric methods according to the analytical Eco-Scale per sample.

Items	Value	Sub total	PPs
Reagents SDS			
Reagent amount	10–100 mL	2	4
Reagent hazard		2	
Instruments			
Energy	˂ 0.1 kWh per sample	0	0
Occupational hazard	Emission of vapors and gases to the air	0	0
Waste	1–10 mL	3	6
No treatment		3	
Total PPs			10
Analytical eco-scale total score from 100			90

**Table 7:** Developed methods in comparison with reported methods.

Method	Drug	Proposed methods		Reported methods		
		UV method	MCR method	Spectrophotometric methods (10)	HPLC method (9)	HPTLC method (9)
Linearity (μg mL^1−^)	BUP	70–900	100–900	50–700	10.0–200.0	5.0–150.0
	MEL	20–120	20–120	1–10	1.0–100.0	1.0–25.0
LOD (μg mL^1−^)	BUP	13.2	22.77	8.86–13.64	3.198	0.48
	MEL	2.39	5.65	0.06–0.31	0.314	0.311
Solvent	0.3%SDS			0.1N HCl*	Methanol*	Methanol*
Analytical eco scale	90			90	73	80

*PP of HCl = 2, PP of methanol = 6.

## Conclusion

This research presents simple and straightforward spectrophotometric methods for simultaneous determination of BUP and MEL in their dosage form (Derivative dual wavelength and MCR-ALS). Derivative dual wavelength is a novel method that enables determination of binary component where the application of dual wavelength is not feasible. MCR-ALS is a chemmometric method that is useful in analysis of multicomponent drug mixtures with overlapped spectra without prior separation. Both methods are simple, accurate, reliable and do not include complicated calculations. The results of accuracy and precision were comparable to those of the reported HPLC method. They are also applicable for other multicomponent drug mixture.

### Supplementary Information

Supplementary Information.

## Data Availability

All data generated or analyzed during this study are included in this published article (and its Supplementary Information files).
