# Supplementary material for: Simultaneous determination of meloxicam and bupivacaine via a novel modified dual wavelength method and an advanced chemometric approach
Source: Sci Rep. 2024 Jan 22;14:1893. doi: 10.1038/s41598-024-51885-z (PMC10803767; doi:10.1038/s41598-024-51885-z)
Supplement: Supplementary file 1 — Supplementary Information. [file 41598_2024_51885_MOESM1_ESM.docx]

| **Simultaneous Determination of Meloxicam and Bupivacaine via a Novel Modified Dual Wavelength Method and Advanced Chemometric Approach**  **Samah F. El-Malla ^1^, Aliaa A. Hamza^1^, Samar H. Elagamy^1^***  1 Department of pharmaceutical analytical chemistry, Faculty of pharmacy, Tanta university, Tanta, Egypt  *samar.elagamy@pharm.tanta.edu.eg |
| --- |
| **Supplementary figures and tables** |

**Figure S1:** UV absorption spectra of mixture 500µg.mL^-1^ of BUP and 50 µg.mL^-1^ MEL in different concentrations of SDS.

Table S1: concentration of BUP and MEL in calibration and validation mixture

| BUP* (µg.mL^-1^) | | MEL*(µg.mL^-1^) |
| --- | --- | --- |
| **Validation set** | 200  500  300  400  75 | 50  80  40  100  25 |
| **Calibration set** | 100  200  200  200  300  300  300  400  500  100  600  600  600  700  700  800  800  800  900  900 | 50  40  20  50  100  30  60  40  50  20  60  20  100  100  20  40  120  20  100  30 |

*Each mixture prepared in triplicate.
